# Bone Marrow Aplasia and Neutropenic Fever Following Azathioprine Dose Escalation in a TPMT-Deficient Patient with Crohn’s Disease and Psoriatic Arthritis—A CARE–Compliant Case

**DOI:** 10.3390/clinpract15060114

**Published:** 2025-06-19

**Authors:** Krzysztof Wroński, Michał Tadeusz Holecki, Natalia Boguszewska, Marzena Skrzypczak-Zielińska, Jerzy Tadeusz Chudek

**Affiliations:** 1 Department of Internal Medicine and Oncological Chemotherapy, Faculty of Medical Sciences in Katowice, Medical University of Silesia in Katowice, 40-029 Katowice, Poland; 2 Department of Internal Medicine and Metabolic Diseases, Faculty of Medical Sciences in Katowice, Medical University of Silesia in Katowice, 40-752 Katowice, Poland; mholecki@sum.edu.pl; 3 Institute of Human Genetics, Polish Academy of Sciences, 60-479 Poznań, Poland; natalia.boguszewska@igcz.poznan.pl (N.B.); marzena.skrzypczak-zielinska@igcz.poznan.pl (M.S.-Z.)

**Keywords:** nucleoside diphosphate-linked moiety X-type motif 15, thiopurine methyltransferase, mesalazine, azathioprine, bone marrow aplasia, neutropenic fever

## Abstract

**Background:** Myelotoxicity, usually manifested by moderate leukopenia (particularly neutropenia), is a well-known adverse drug reaction to azathioprine (AZA) therapy. Thiopurine methyltransferase (*TMPT*) and nucleoside diphosphate-linked moiety X-type motif 15 (*NUDT15)* genotyping are not routinely performed in patients starting AZA therapy due to their low cost-effectiveness. Additionally, the concomitant use of xanthine oxidase inhibitors and 5-aminosalicylates may slow the metabolism of 6-mercaptopurine. **Case Description:** We describe a case of a 26-year-old Caucasian man with Crohn’s disease and psoriatic arthritis treated with mesalazine and AZA (100 mg daily) who developed prolonged bone marrow aplasia and neutropenic fever after increasing the daily dose of AZA from 100 to 150 mg (from 44 to 66 mg/m^2^), without frequent total blood count monitoring. Discontinuation of AZA, multiple transfusions of red blood cells and platelet concentrate, filgrastim, empirical antibiotic therapy, and antiviral and antifungal prophylaxis were obtained after 11 days complete recovery of bone marrow aplasia. **Methods:** Genomic DNA genotyping of coding regions of *TPMT* (exons 2–9) and *NUDT15* (exons 1–3). **Results:** Heterozygous alleles in the untranslated region (c.460G>A and c.719A>G) associated with TPMT deficiency and a benign variant (c.*7G>A) in the 3′-UTR of *NUDT15* with no effect on enzyme activity were found. **Conclusions:** This case highlights the importance of monitoring the total blood count frequently during the first weeks of treatment with moderate-to-high doses of AZA. Furthermore, the interaction between AZA and mesalazine may play a significant role in the development of prolonged bone marrow aplasia.

## 1. Introduction

Myelotoxicity and hepatotoxicity are well-known adverse drug reactions (ADRs) to azathioprine (AZA) therapy. Leukopenia (particularly neutropenia), usually moderate, is the most common manifestation of bone marrow injury, which occurs with an estimated incidence of 4.5–34% in patients with inflammatory bowel diseases (IBD) treated with AZA [1]. The daily dose of the medication, certain co-medications, older age, and genetically determined degradation of the active metabolite of AZA—6-mercaptopurine (6-MP) are established risk factors for leukopenia [2]. Despite this, routine clinical practice typically involves monitoring only the total blood count (TBC) and liver enzymes during AZA treatment.

Eight variant alleles of thiopurine methyltransferase (*TPMT*) have been reported to reduce the ability of TPMT to metabolize 6-MP [3]. *TPMT* genotypes result in a trimodal distribution of the enzyme activity in red blood cells (RBCs): low (<5.0 U/mL RBCs) with a frequency of 0.3%; intermediate (5.0–13.7 U/mL RBCs) with a frequency of 11.1%; and normal or high (13.8–25.1 U/mL RBCs) with a frequency of 88.6% in the general Caucasian population [4].

Patients treated with moderate-to-high doses of AZA and who have low or intermediate TPMT activity are at risk of myelosuppression episodes caused by excessive accumulation of the active thiopurine metabolites [5,6]. However, *TMPT* genotype should not be considered the only predictor of AZA toxicity. Recently, the nucleoside diphosphate-linked moiety X-type motif 15 (*NUDT15*) genotype at codon 139 was shown to be a stronger predictor of severe leukopenia than the *TMPT* genotype in Japanese patients with IBD [7]. In addition, combination therapy, especially with xanthine oxidase inhibitors (e.g., allopurinol and febuxostat) and 5-aminosalicylates (5-ASA), may slow 6-MP metabolism [8]. It has been found that mesalazine (5-ASA), and its primary metabolite, N-acetyl-5-ASA, reversibly inhibit the activity of TPMT in vitro [9]. As many as one in ten Crohn’s disease (CD) patients in the Mayo Clinic cohort receiving 5-ASA with AZA developed clinically significant leukopenia (<3.0 × 10^6^/L) [9]. In a Chinese cohort, 32.8% of 64 patients treated with AZA and 5-ASA developed leukopenia (<3.5 × 10^6^/L), and three patients had neutropenia (<1.5 × 10^6^/L) with infections [2].

## 2. Case Description

A 26-year-old Caucasian man, with a 3-year history of CD, confirmed by histological examination of colon biopsy, and psoriatic arthritis (PA) was transferred from the Department of Internal Medicine due to prolonged agranulocytosis (WBC 1.64 × 10^3^/µL, reference range—Ref 4.0–10.0; neutrophil count 0.13 × 10^3^/µL, Ref 2.5–5.0; PLT 50 × 10^3^/µL, Ref 130–400; HGB 6.0 g/dL, Ref 13.5–16.5) and neutropenic fever. Fatigue and fever appeared one week before hospital admission. During hospitalization before referral, he received multiple transfusions of red blood cells and platelet concentrate, and for 3 days, was treated with filgrastim, a granulocyte-colony stimulating factor (G-CSF), but without the expected effect.

On admission, the patient still presented with fatigue, mild diarrhea, joint pain, and pruritus. Physical examination revealed pale skin and a rash covering the torso and arms. No fever or other symptoms of infection were observed. The laboratory tests confirmed normocytic anemia (HGB 8.2 g/dL; Ref 11.2–15.8), leukopenia (WBC 0.87 × 10^3^/µL; Ref 4.0–10.0) with severe neutropenia (0.06 × 10^3^/µL; Ref 1.6–6) and thrombocytopenia (37.0 × 10^3^/µL; Ref 130–400), serum creatinine level was slightly elevated (131.6 µmol/L; Ref 63.6–110.5), as well as lactate dehydrogenase (LDH) activity (352 IU/L; Ref 125–220). The patient (body weight 98.6 kg, body surface 2.26 m^2^) recently had his AZA dose increased from 100 to 150 mg daily (from 1.01 to 1.52 mg/kg body weight; from 44 to 66 mg/m^2^) in addition to continued therapy with 3 g of 5-ASA and 10 mg of prednisone daily, due to persistent activity of CD. The last TBC was performed approximately 2 months before hospital admission, before AZA dose escalation. AZA was added to the ongoing 5-ASA treatment approximately 8 months before the adverse event occurred.

**Figure 1:**
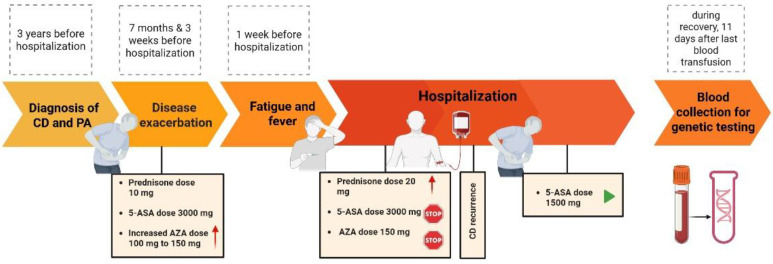
Timeline of events. Created in BioRender. Skrzypczak-Zielinska, M. (2025) https://BioRender.com/kb2ucmi. (accessed on 31 May 2025).

Both AZA and 5-ASA were discontinued, and the prednisone dose was increased to 20 mg daily (Figure 1). Regardless of the antibiotic, antiviral, and antifungal prophylaxis used, and the continuous administration of filgrastim, deterioration of the general condition with fever and a significant increase in inflammatory parameters was observed (C-reactive protein—CRP increased from 22 mg/L (Ref < 5) to 262 mg/L within two days). After empirical modification of antibiotic therapy (ciprofloxacin monotherapy was replaced with levofloxacin and amikacin combination), the symptoms of infection subsided. From the third day, the CRP concentration began to decrease.

Following the initial observation of a slight decrease in hemoglobin levels, we noted a steady increase without the need for red blood cell transfusions. Due to symptomatic thrombocytopenia (hemorrhagic diathesis), two units of platelet concentrate were transfused. Neutropenia persisted for eight days. Due to the recurrence of CD symptoms, the patient restarted 5-ASA therapy at a dose of 1500 mg/day without symptoms of myelotoxicity. Treatment with 5-ASA was reintroduced due to its previous good tolerance without exacerbation of psoriasis, and further therapeutic decisions were postponed until full bone marrow recovery.

On the day of discharge, moderate thrombocytopenia (PLT 38 × 10^3^/µL) and anemia (HGB 10.0 g/dL) persisted, but the neutrophil count was normal (2.20 × 10^3^/µL). According to clinical assessment, severe bone marrow aplasia is most likely related to drug interactions between AZA and 5-ASA. However, a genetic disorder in the metabolism of 6-MP could not be excluded. Therefore, a blood sample was sent for genetic testing before discharge.

## 3. Materials and Methods

Genomic DNA was isolated from the peripheral blood using a guanidine isothiocyanate solution and stored at 4 °C in a TE buffer containing 1 mM EDTA and 10 mM Tris-Cl. Amplification of the *TPMT* coding region (exons 2–9) was performed as previously described [10], except for the modified primers for exon 4, which had the following sequences: 5′CCTGCATGTTCTTTGAAACCCTATGAA3′-forward and 5′GAGGTAAAACTTTTGTGGGGATATGGA3′-reverse, obtaining an amplicon length of 508 bp.

Primers for the amplification of the *NUDT15* coding region (exons 1–3) were designed using the Primer3-BLAST design tool (National Library of Medicine, Bethesda, MD, USA), and their sequences are presented in Table 1. Polymerase chain reaction (PCR) was performed in a total volume of 25 μL with 100 ng of genomic DNA, 2.5 μL of 10-times concentrated buffer, 1.5 mM MgCl_2_, 200 μM deoxyribonucleotide triphosphate, 0.15 μM of each primer, and 1 unit of FIREPol DNA Polymerase (Solis BioDyne, Tartu, Estonia). Amplification was performed using a T100 Thermal Cycler (Bio-Rad, Hercules, CA, USA). The PCR program started with an initial denaturation at 95 °C for 4 min, followed by 40 cycles of denaturation at 94 °C for 30 s, annealing a temperature of 66 °C was gradually reduced (1 °C /cycle) for 15 cycles and maintained at 51 °C for the next 25 cycles, for 30 s, and extension at 72 °C for 60 s, as well as a final extension at 72 °C for 7 min. Sanger sequencing was performed in both directions on an Applied Biosystems 3500 Genetic Analyzer (Thermo Fisher Scientific, Waltham, MA, USA) using a BigDye Terminator v3.1 Cycle Sequencing Kit (Thermo Fisher Scientific), according to the manufacturer’s instructions. DNA sequences were analyzed using Sequencing Analysis Software (Thermo Fisher Scientific).

## 4. Results

**Figure 2:**
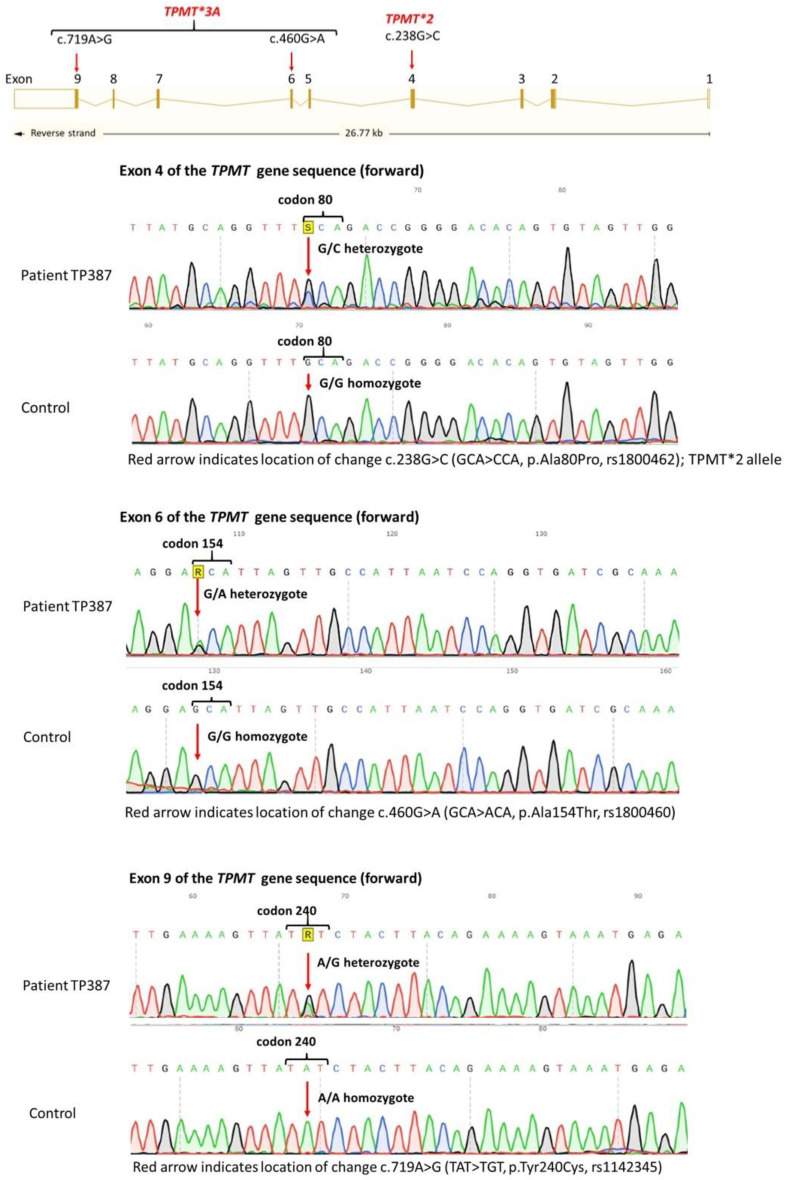
DNA sequencing of *TPMT* showing heterozygous allele TPMT*2 (c.238G>C—p.Ala80Pro, rs1800462) and TPMT*3A (haplotype of two variants: c.460G>A—p.Ala154Thr, rs1800460, and c.719A>G—p.Tyr240Cys, rs1142345) and location of these alleles in *TPMT*.

DNA sequencing revealed a compound heterozygous allele TPMT*2 (c.238G>C—p.Ala80Pro, rs1800462) and TPMT*3A (haplotype of two variants: c.460G>A—p.Ala154Thr, rs1800460, and c.719A>G—p.Tyr240Cys, rs1142345) (Figure 2), which were described in databases (The Human Gene Mutation Database—HGMD, Ensembl) and the literature as alleles responsible for the deficiency of TPMT enzyme activity [8]. These co-occurring variants in a single patient create the allele system (diplotype) TPMT*2/*3A (c.[238G>C];[460G>A;c.719A>G]), which determines the deficit of TPMT enzyme activity and, as a result, poor 6-MP metabolism (poor metabolizer).

The TPMT*2 allele is relatively rare in the European population, with a frequency of 0.6% based on the 1000 Genomes Project data, Phase 3 (https://www.ensembl.org/Homo_sapiens/Variation/Population?db=core;r=6:18143224-18144224;v=rs1800462;vdb=variation;vf=406243412 accessed on 21 October 2024). However, the TPMT*3A allele, which is a haplotype comprising two variants, c.460G>A and c.719A>G, is present at a frequency of 2.6–5.7% in Europeans, including 3.28% in the Polish population [10].

Genotyping of *NUDT15* revealed a benign variant, rs61973267 (c.*7G>A), in the 3′ UTR region, which did not affect enzyme activity.

## 5. Discussion

Myelotoxicity with mild leukopenia is a well-known ADR to AZA therapy, especially when combined with 5-ASA. To our knowledge, no neutropenic fever case associated with bone marrow aplasia resulting from such an interaction has been previously reported. In the present case, neutropenic fever persisted for 11 days despite the administration of filgrastim. This was caused by the lack of TBC monitoring in the first weeks after increasing the AZA dose from 100 mg to 150 mg daily in a poor 6-MP metabolizer. The *TPMT* genotype (TPMT*2/*3A diplotype), which determines TPMT enzyme deficiency, was more critical than drug interactions with AZA and 5-ASA. The prescribed moderate dose of AZA (66 mg/m^2^) in a poor TPMT metabolizer can cause leukopenia itself. However, drug interaction probably enhanced this effect and played a significant role in long-lasting bone marrow aplasia. As shown by Morikubo et al., mesalazine administration caused TPMT inhibition in patients with ulcerative colitis [9].

Lowry et al. evaluated the incidence of leukopenia in patients concurrently receiving 5-ASA and AZA with monitored metabolite concentrations and TPMT activity [10]. The study confirmed that, in most cases, the simultaneous use of 5-ASA and AZA is strongly associated with the occurrence of leukopenia. However, *TPMT* and *NUDT15* genotyping were not performed in this study.

According to de Graaf et al. [11], the co-administration of AZA and 5-ASA may increase the concentration of 6-MP metabolites in the plasma, raising the risk of myelotoxicity. Moreover, genetic variants associated with reduced TPMT activity are considered significant risk factors for the development of AZA-related myelotoxicity [12].

More severe myelotoxicity has been sporadically reported in patients treated with AZA and 5-ASA by the literature. In the case of a 24-year-old woman described by Barnhoorn et al., a decrease in neutrophil count (1.88 × 10^3^/µL) was observed as a result of switching 5-ASA compounds (from 4 g of Salofalk^®^ granulate to 4 g of Pentasa^®^ granulate 8 weeks before presentation) following long-term co-administration of AZA and 5-ASA. However, in this case, anemia was the main symptom of myelotoxicity [13]. In addition, *TPMT* and *NUDT15* genotyping were not performed in this study.

There are few other reports of severe bone marrow aplasia (aplastic anemia) in patients treated with AZA for rheumatological diseases [14,15]. Montgomery et al. described a patient with rheumatoid arthritis who was hospitalized for acquired aplastic anemia related to TPMT deficiency during AZA therapy [14]. Yeter et al. reported aplastic anemia within 4 weeks of AZA therapy, which resulted in death unrelated to abnormal TPMT activity in a young woman with systemic lupus erythematosus [15]. In the present case, *NUDT15* genotyping was not performed.

Contrary to the reviewed cases of severe myelotoxicity induced by AZA, we performed genetic testing of both *TPMT* and *NUDT15*, which is still not routinely performed in our country in patients starting the treatment. Moreover, the interaction with 5-ASA is poorly recognized by clinicians. In our case, frequent total blood count monitoring after AZA dose escalation was ignored by the gastrologist, leading to the late diagnosis of myelotoxicity.

Implementing the Clinical Pharmacogenetics Consortium guidelines on AZA dosing based on *TPMT* and *NUDT15* genotypes [8] requires prior testing of *TPMT* and *NUDT15* in patients with a starting dose of 75 mg/m^2^/day or higher. Dose reduction is recommended for poor metabolizers of mercaptopurine and should be considered for intermediate metabolizers. However, there is still a risk that some variants affecting mercaptopurine metabolism may not be detected using the currently used assays. The genotyping approach is limited to detecting known variants within the coding regions of *TPMT* and *NUDT15* genes. It does not capture rare or novel variants, copy number variations, functional consequences outside these regions, or epigenetic factors. Therefore, complementary testing, such as TPMT and NUDT15 enzymatic activity assays, may provide additional clinically relevant information in cases of inconclusive or discordant genotype–phenotype correlations. While enzymatic testing was not feasible in our study due to limited sample availability, it remains a valuable tool, particularly when genotyping alone is insufficient for reliable phenotype prediction. Currently, the availability of genotyping is limited in healthcare systems, and the procedure itself is time-consuming. Moreover, the cost-effectiveness of combined screening for *TPMT* and *NUDT15* defective alleles is relatively high (980 EUR/patient) and is not realistic for the majority of Caucasian populations. As estimated, genotyping of 10,000 patients would avoid 43 episodes of severe myelosuppression [16]. Until genotyping becomes cheaper and more accessible, we must carefully monitor TBC in patients starting moderate-to-high doses of AZA therapy and pay more attention to drug interactions.

Polish guidelines from 2013 did not mention how to monitor the safety of AZA therapy [17]. In 2023, new guidelines were published that recommend monitoring based on assessing the blood count, alanine aminotransferase, and creatinine levels every two weeks during the first two months of therapy. Monitoring of 6-tioguanine metabolites is considered optional [18]. These guidelines are more detailed than those of the World Gastroenterology Organization, specifying the blood count monitoring after increasing the thiopurine dose from 50 mg to the full dose if TPMT measurement is unavailable [19].

Laboratory techniques for monitoring thiopurine therapy are under development. Recently, measurement of DNA-incorporated thioguanine (DNA-TG) levels has been introduced as a potentially more appropriate method for predicting clinical response and toxicities such as leukopenia [20]. However, until this is implemented, blood count monitoring remains the most widely available method of monitoring thiopurine therapy.

In the coming years, the use of AZA in the treatment of inflammatory bowel disease is expected to decrease due to the rapid improvement of biological therapy and its increasing use in patients with moderate-to-severe clinical courses, in line with current guidelines [21]. This will decrease the risk of AZA myelotoxicity and will eliminate the need for genetic testing of *TPMT* and *NUDT15,* at least in wealthy countries.

The reintroduction of 5-ASA during the bone marrow regeneration process was not supported by guidelines, and could be considered suboptimal, as the therapy was not fully effective in treating CD in our patient. Initiation of treatment with one of the registered biological agents was an alternative, but the decision was postponed until the patient’s bone marrow had fully recovered.

## 6. Conclusions

In conclusion, in our case, severe, prolonged bone marrow aplasia was caused by the lack of TBC monitoring after increasing the dose of AZA in a poor mercaptopurine metabolizer co-treated with 5-ASA for psoriatic arthritis coexisting with Crohn′s disease.

We emphasize the need for frequent monitoring of the total blood count during the first weeks of treatment with moderate-to-high doses of AZA.

## Figures and Tables

**Table 1:** Sequences of primers used for amplification of the *NUDT15* gene coding region.

	Primer Sequence (5′→3′)	Chromosome Coordinates GRCh38/hg38	Amplicon (bp)
Exon 1	F: CAAAGCACAACTGTAAGCGACT R: GAAAGACCCAGCTAGCAAAGAC	chr13:48037494 + 48038124	631
Exon 2	F: CGGCCTTCCAAAAGATTACA R: TGATCTAATCACCTCCCAAGG	chr13:48040685 + 48041309	625
Exon 3	F: AAGCAAATGCAAAGCATCAC R: GGCTGAAAGAGTGGGGGATA	chr13:48045510 + 48045959	450

F—forward, R—reverse.

## Data Availability

The raw data supporting the conclusions of this article will be made available by the authors on request.

## Abbreviations

The following abbreviations are used in this manuscript:

ARD	adverse drug reaction
5-ASA	mesalazine
AZA	azathioprine
DNA-TG	DNA-incorporated thioguanine
IBD	inflammatory bowel diseases
6-MP	6-mercaptopurine
NUDT15	nucleoside diphosphate-linked moiety X-type motif 15
TPMT	thiopurine methyltransferase
